# Chinese Herbal Medicines Have Potentially Beneficial Effects on the Perinatal Outcomes of Pregnant Women

**DOI:** 10.3389/fphar.2022.831690

**Published:** 2022-06-06

**Authors:** Hsuan-Shu Shen, Wei-Chuan Chang, Yi-Lin Chen, Dai-Lun Wu, Shu-Hui Wen, Hsien-Chang Wu

**Affiliations:** ^1^ Department of Chinese Medicine, Hualien Tzu Chi Hospital, Buddhist Tzu Chi Medical Foundation, Hualien, Taiwan; ^2^ School of Post-baccalaureate Chinese Medicine, Tzu Chi University, Hualien, Taiwan; ^3^ Sports Medicine Center, Hualien Tzu Chi Hospital, Buddhist Tzu Chi Medical Foundation, Hualien, Taiwan; ^4^ Department of Medical Research, Buddhist Tzu Chi General Hospital, Hualien, Taiwan; ^5^ Department of Obstetrics and Gynecology, Taipei Tzu Chi Hospital, Buddhist Tzu Chi Medical Foundation, New Taipei City, Taiwan; ^6^ Department of Public Health, College of Medicine, Tzu Chi University, Hualien, Taiwan; ^7^ Department of Chinese Medicine, Taipei Tzu Chi Hospital, Buddhist Tzu Chi Medical Foundation, New Taipei City, Taiwan

**Keywords:** preterm labor, threatened miscarriage, tocolytic treatment, Chinese herbal products, National health insurance research database

## Abstract

**Introduction:** Tocolytic treatment is beneficial to pregnant women with a risk of premature labor or miscarriage. However, previous reports have shown that progestogen might not be effective and ritodrine may increase the risk of maternal vascular-related diseases. Chinese herbal products (CHP) are used as alternative therapies for pregnant women. The goal was to evaluate the efficacy of combined tocolytic therapy and CHP therapy in pregnancy outcomes for pregnant women in Taiwan.

**Materials and Methods:** We conducted a retrospective cohort study based on the National Health Insurance Research Database. A total of 47,153 pregnant women treated with tocolytics aged 18–50 years from 2001 to 2015 were selected from two million random samples. According to the medical use of tocolytics and CHP, we divided the users into two groups: western medicine (WM) only (n = 40,961) and WM/CHP (n = 6,192) groups. A propensity score (PS)-matched cohort (6,192 pairs) was established based on baseline confounders. All participants were followed up to perinatal outcomes. Conditional logistic regression analysis was used to examine the effects of CHP use on the odds of miscarriage and preterm birth.

**Results:** The adjusted odds ratio (OR) for premature birth in the WM/CHP group (n = 411, 6.64%) was significantly lower than in the WM group (n = 471, 7,61%) (0,86, 95% confidence interval [CI], 0.74–0.99). Further subgroup analysis based on the usage of formulae that activate blood and remove stasis or purgative formulae, the adjusted OR of preterm birth of those using these formulae was significantly lower in the WM/CHP group (n = 215, 6.32%) than that in the WM group (n = 265, 7.77%) (OR: 0.79, 95% CI: 0.65–0.96).

**Conclusion:** We found that the combination of CHP and tocolytics can be beneficial to pregnant women in the prevention of premature birth. Further research is required to investigate causal relationships.

## 1 Introduction

The use of tocolytics to prevent or eliminate uterine contractions occurring before 34 weeks of gestation has been the major strategy of obstetric management of threatened preterm labor for many years (Hubinont and Debieve, 2011). Preterm labor is the major cause of perinatal mortality and morbidity in high-income countries and constitutes a huge economic burden on the healthcare system (Blencowe et al., 2013). Administration of tocolytics can reduce the intensity and frequency of uterine contractions to prolong pregnancy, thereby providing time for further intrauterine maturation and interventions that may improve infant outcome (Dehaene et al., 2017).

A met-analysis of women with acute preterm labor found that these drugs were more effective than placebo/control in delaying delivery for 48 h and 7 days, but not for delaying delivery until 37 weeks (Haas et al., 2009). Many different agents have been used for tocolytic therapy to suppress uterine contractions, but a standard first-line drug has not emerged. Currently, drugs commonly used for tocolytic therapy include progesterone, β-agonists, calcium channel blockers, and oxytocin receptor antagonists (Younger et al., 2017; Hanley et al., 2019). In Taiwan, a perspective survey of tocolytic treatment for preterm birth prevention had revealed that nearly 60% of obstetric specialists prescribed ritodrine as the first-line medication, whether in oral or intravenous form. The second choice was nifedipine, and only 6% of. obstetrician gynecologists prescribed atosiban, a self-paid medication in Taiwan, as first-line tocolytics (Lee et al., 2022). However, the Pharmacovigilance Risk Assessment Committee (PRAC) of the European Medicines Agency (EMA) issued a warning in October 2013, stating that β-adrenergic receptor agonists (such as ritodrine) should only be used at gestational age 22–37 weeks and the duration of administration should not exceed 48 h. Long-term use of β-adrenergic receptor agonists has a high cardiovascular risk, which may outweigh its benefits (EMA, 2013). Therefore, the safety and efficacy of tocolytics for the treatment of spontaneous preterm labor or threatened miscarriage during pregnancy is of concern to pregnant women and physicians (Lamont and Jorgensen, 2019).

In addition to western medicine (WM), traditional Chinese medicine (TCM) treatment is another option. Chinese herbal medicine (CHM) has a long history of preventing miscarriage and premature birth. A review of relevant literature shows that compared with WM alone, the combined use of TCM and WM tocolytic treatments exhibits superior results and fewer side effects. The most frequently used formula is“Shou Tai Pill,” and it was thought to enhance the function of “Kidney” and regulate the “Qi” in the human body, thus improving the health condition of mothers and benefiting the fetus (Li et al., 2012; Li et al., 2014). The use of CHM during pregnancy or postpartum has been analyzed and reported in the literature (Chuang et al., 2009; Yeh et al., 2009). However, the safety and effectiveness of CHM use in pregnant women with signs of miscarriage or premature birth have not been further evaluated.

The National Health Insurance (NHI) research database (NHIRD) in Taiwan has long-term tracking data and an excellent drug registration process, which allows tracking of the efficacy and safety of Chinese herbal products (CHP) from retrospective cohort data. The purpose of this study was to use the NHIRD to explore the combined use of tocolytic drugs and CHP by pregnant women in Taiwan and evaluate their effects on birth outcome (including preterm delivery and miscarriage). The effectiveness of WM tocolytic drugs was also compared with that of their combined use with CHM preparations.

## 2 Materials and Methods

### 2.1 Data Resources

Taiwan launched a single-payer NHI Program in 1995 that covers almost the entire population. The NHI program database contains registration files and original claim data for reimbursement, while data in the NHIRD derived from the NHI program are released for research purposes. The data used in this study was a longitudinal health insurance database 2000 (LHID 2000) comprising medical claim data of 2,000,000 beneficiaries randomly sampled from the 2000 Registry for beneficiaries of the NHIRD.

The randomly sampled beneficiaries exhibited no significant differences in age, sex, birth year, and other basic characteristics from the general population. We used LHID 2000 data from 2001 to 2015, including inpatient and outpatient visits and medications, and the International Classification of Disease-Clinical Modification, ninth revision (ICD-9-CM) was used to define the disease in this study. The study was approved by the research ethics committee of Buddhist Tzu Chi General Hospital, Hualien.

### 2.2 Study Samples and Measured Variables

**FIGURE 1:**
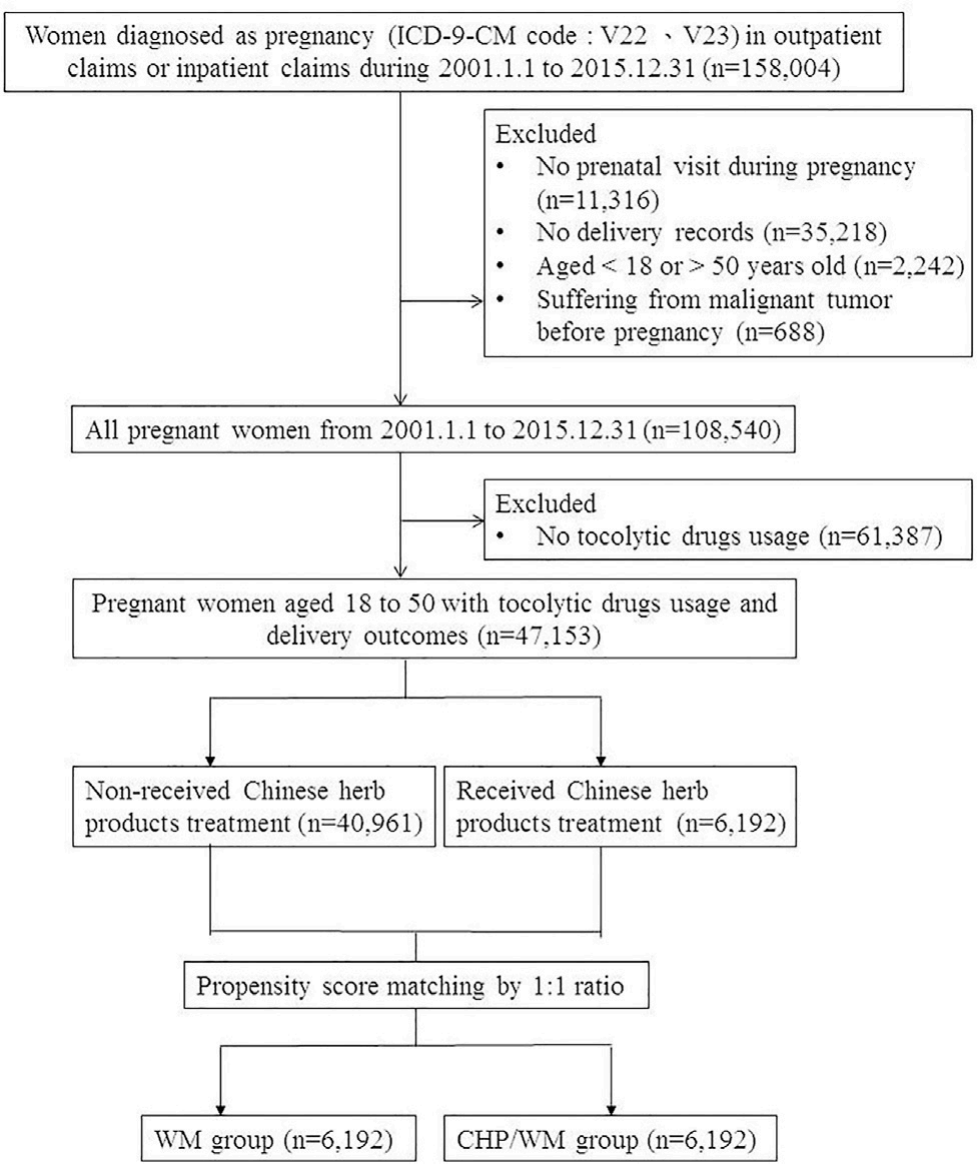
Flow chart of this study design.

This was a retrospective cohort study based on data from the LHID 2000 collected between 1 Jan 2001 to 31 Dec 2015. There were 158,004 women with the diagnosis of pregnancy (ICD-9-CM codes:V22 and V23) during outpatient or inpatient visits. The exclusion criteria were as follows: 1) no prenatal visits (n = 11,316), 2) no birth outcome information including term birth (ICD-9-CM code: V27.0, V27.2, V27.3, V27.5, V27.6, V30-V37, V39, 650), preterm birth (ICD-9-CM code: 765.1), miscarriage (ICD-9-CM code: 632; ICD_OP_CODE:690, 695, 750), and stillbirth (ICD-9-CM code: V27.1, V27.4, V27.7, 656.4) (n = 35,218), 3) aged <18 or >50 years old (n = 2,242), 4) malignant tumor before pregnancy (n = 688), and 5) did not use any tocolytic drugs (n = 61,387). Based on the inclusion and exclusion criteria, we selected a total of 47,153 eligible pregnant women in our study cohort (Figure 1).

Pregnant women who had TCM outpatient visits along with CHP prescription records because of obstetrics-related disease (ICD-9-CM code: 610-677 or A code: A370-A394) in parallel to their tocolytic treatment were allocated to the WM/CHP group. Pregnant women who only received tocolytic treatment were assigned to the WM group. The index date of both groups was the estimated date of their last menstrual period and the end date was the date when labor ended. Before matching, there were 6,192 and 40,961 women in WM/CHP and WM groups, respectively.

### 2.3 Primary Outcome

The primary outcome was the labor results during the follow-up period including term birth, preterm birth, miscarriage, and stillbirth based on the ICD-9-CM code diagnosed by obstetrician-gynecologists. (ICD-9-CM code was described in the previous Section 2.2).

### 2.4 Characteristics, Comorbidities, and Drug

The following sociodemographic variables were collected: maternal age, year of pregnancy, first TCM outpatient visit (before or after pregnancy), frequency of prenatal visits, first prenatal visit, and pregnancy duration. The exact date of the last menstrual period was not available for the participants, so we indirectly deduced it from the date of the first prenatal visit. Pregnant women are offered 10 free prenatal visits during gestation in Taiwan. Each free prenatal visit with its corresponding sequence number from 41 to 50 represented specific gestational times, with number 41 representing the first free prenatal visit of the first trimester. The last menstrual period was determined by counting back 90 days from the sequence number 41. Duration of pregnancy was defined as the period between the estimated date of last menstrual period and the date of delivery.

Moreover, we included the comorbidities recommended by Bateman et al. (2013) such as severe preeclampsia/eclampsia (ICD-9-CM: 642.5, 642.6), mild preeclampsia (ICD-9-CM: 642.4), gestational hypertension (ICD-9-CM: 642.3), unspecified preeclampsia (ICD-9-CM: 642.7), chronic congestive heart failure (ICD-9-CM: 428.22, 428.23, 428.32, 428.33, 428.42, 428.43), congenital heart disease (ICD-9-CM: 745–747.4, 648.5), chronic ischemic heart disease (ICD-9-CM: 412-414), cardiac valvular disease (ICD-9-CM: 394-397, 424), pulmonary hypertension (ICD-9-CM: 416.0, 416.8, 416.9), preexisting hypertension (ICD-9-CM: 401-405, 642.0–642.2), sickle cell disease (ICD-9-CM: 282.4, 282.6), systemic lupus erythematosus (ICD-9-CM: 710.00), human immunodeficiency virus (ICD-9-CM: 042, V08), placenta previa (ICD-9-CM: 641.0, 641.1), chronic renal disease (ICD-9-CM: 581-583, 585, 587, 588, 646.2), asthma (ICD-9-CM: 493), preexisting diabetes mellitus (ICD-9-CM: 250, 648.0), multiple gestation (ICD-9-CM: V27.2-V27.8, 651), drug abuse (ICD-9-CM: 304, 305.2–3.05.9, 648.3), alcohol abuse (ICD-9-CM: 291, 303, 305.0), and previous cesarean delivery (ICD-9-CM: 654.2) (Bateman et al., 2013).

These diseases were determined by at least two outpatient visits or one inpatient visit at least 1 year before enrollment. We also considered the medication and cumulative usage days, including progestins (ATC code: G03DA04, G03DA03, G03DC01), ritodrine (ATC code: G02CA01), and nifedipine (ATC code: C08CA05). In addition, to fairly compare WM/CHP group and WM group, we adopted propensity score (PS) matching at a ratio of 1:1 of WM/CHP group to the matched WM group (Figure 1). PS was calculated using logistic regression with maternal age, pregnancy year, TCM outpatient visits before the half year of pregnancy, and comorbidity index at enrollment. The WM/CHP and WM groups were matched using the greedy matching algorithm of the log odds ratio (logit) of the PS within a caliper of 0.2 of the standard deviation of the logit of the PS.

### 2.5 Statistical Analyses

Continuous variables are reported as means and standard deviation, whereas categorical variables are reported as frequencies and percentage. We used an independent sample *t*-test or Chi-square test to compare the means or proportions of sample characteristics of the two groups. Furthermore, we used a conditional logistic regression analysis to examine the effect of CHP use on the outcome of pregnant women, controlling for potential confounding factors including maternal age at delivery, categories of tocolytic drugs used, and cumulative days of tocolytic drug use. In addition, to examine the safety of the different types of CHP used, we stratified pregnant women into two groups: those who used 1) products for activating blood and removing stasis or purgatives and 2) products that did not have these properties. The mechanisms of activating blood and removing stasis formulae or purgatives formulae were dilating blood vessels and inhibiting platelet aggregation. The most commonly used formula, Gui-Zhi-Fu-Ling-Wan, possesses significant spasmolytic effects on uterine tetanic contractions (Sun et al., 2016). Accordingly, activating blood and removing stasis formulae or purgatives formulae were believed to have the potential to prevent miscarriage or preterm birth. The *p* value <0.05 was considered statistically significant. Data were analyzed using SAS version 9.3 (SAS Institute, Inc., Cary, NC, Unite States).

## 3 Results

There were 108,540 pregnant women in our study period, and we enrolled 47,153 pregnant women in the cohort according to the inclusion and exclusion criteria, consisting of 6,192 who received CHP because of obstetrics-related diseases with a CHP utilization rate of 13.1%. The mean age was 29.75 years old and >99% of the sample population had their first prenatal visit in the first trimester. The estimated mean duration of pregnancy was 284 days. Before PS matching, the WM/CHP group tended to be older and a higher proportion experienced threatened abortion, so we conducted PS-matched to balance the baseline characteristics between the two groups. After PS matching, there were still differences in the usage of medications. In terms of progestins, compared to pregnant women in the WM group, more pregnant women in the WM/CHP group received progestins treatment (68.44% vs 72.06%, respectively). Among them, more than 95% women treated with progestins in the first trimester. Regarding tocolytic drugs, less than 3% pregnant women were prescribed Ritodrine or Nifedipine in their first trimester. Among women using Ritodrine or Nifedipine, over 90% pregnant women were prescribed Ritodrine or Nifedipine in their second or third trimester both in WM group and WM/CHP group. In addition, a higher percentage of women in the WM/CHP group received more than one tocolytic drug than those in the WM group (28.37% vs 25.89%, respectively). As to CHP, nearly 78% of pregnant women in the WM/CHP group received TCM treatment in the first trimester and the mean cumulative usage days of CHP was 23.72 days (Table1).

**TABLE 1:** Characteristics of pregnant women classified according to the use of Chinese herbal products.

	Before Match			After PS Match		
	WM/CHP (n = 6,192)	WM (n = 40,961)	*p*-value	WM/CHP (n = 6,192)	WM (n = 6,192)	*p*-value
PS score	0.21 (0.11)	0.12 (0.10)	<0.001*	0.21 (0.11)	0.21 (0.11)	0.999
Maternal age at delivery	29.75 (4.32)	28.56 (4.65)	<0.001*	29.75 (4.32)	29.75 (4.32)	0.998
Year at pregnancy			<0.001*			1.000
2001	202 (3.26)	3,051 (7.45)		202 (3.26)	205 (3.31)	
2002	220 (3.55)	2,612 (6.38)		220 (3.55)	218 (3.52)	
2003	283 (4.57)	2,537 (6.19)		283 (4.57)	289 (4.67)	
2004	293 (4.73)	2,480 (6.05)		293 (4.73)	290 (4.68)	
2005	296 (4.78)	2,603 (6.35)		296 (4.78)	297 (4.80)	
2006	332 (5.36)	2,672 (6.52)		332 (5.36)	333 (5.38)	
2007	384 (6.20)	2,767 (6.76)		384 (6.20)	385 (6.22)	
2008	431 (6.96)	2,600 (6.35)		431 (6.96)	422 (7.14)	
2009	442 (7.14)	2,437 (5.95		442 (7.14)	442 (6.82)	
2010	447 (7.22)	2,703 (6.60)		447 (7.22)	445 (7.19)	
2011	653 (10.55)	3,556 (8.68)		653 (10.55)	662 (10.69)	
2012	644 (10.40)	3,320 (8.11)		644 (10.40)	642 (10.37)	
2013	617 (9.96)	3,354 (8.19)		617 (9.96)	614 (9.92)	
2014	766 (12.37)	3,517 (8.59)		766 (12.37)	766 (12.37)	
2015	182 (2.94)	752 (1.84)		182 (2.94)	182 (2.94)	
Comorbidity index ≥1	1,139 (18.39)	5,972 (14.58)	<0.001*	1,139 (18.39)	1,121 (18.10)	0.675
TCM visit within half years before pregnancy	4,441 (71.72)	12,952 (31.62)	<0.001*	4,441 (71.72)	4,441 (71.72)	1.000
Threatened abortion	5,442 (87.89	35,149 (85.81)	<0.001*	5,442 (87.89)	5,380 (86.89)	0.093
1st trimester	4,807 (88.33)	29,914 (85.11)	<0.001*	4,807 (88.33)	4,642 (86.28)	0.006*
2nd trimester	358 (6.58)	2,904 (8.26)		358 (6.58)	421 (7.83)	
3rd trimester	277 (5.09)	2,331 (6.63)		277 (5.09)	317 (5.89)	
Pregnancy duration	284.8 (33.76)	282.6 (32.08)	<0.001*	284.8 (33.76)	283.6 (31.81)	0.043
Total prenatal visits	10.82 (2.23)	10.62 (2.26)	<0.001*	10.82 (2.23)	10.81 (2.14)	0.951
First prenatal visit in **1**st trimester	6,142 (99.19)	40,423 (98.69)	<0.001*	6,142 (99.19)	6,138 (99.13)	0.694
Types of Tocolytics						
Progestin usage	4,686 (75.68)	28,807 (70.33)	<0.001*	4,686 (75.68)	4,443 (71.75)	<0.001*
**1**st **trimester**	4,462 (72.06)	27,464 (67.05)	<0.001*	4,462 (72.06)	4,238 (68.44)	<0.001*
**2**nd **trimester**	592 (9.56)	2,825 (6.90)	<0.001*	592 (9.56)	466 (7.53)	<0.001*
**3**rd **trimester**	49 (0.79)	243 (0.59)	0.064	49 (0.79)	33 (0.53)	0.076
Progestin usage days	10.69 (12.09)	8.46 (9.24)	<0.001*	10.69 (12.09)	9.21 (10.01)	<0.001*
Ritodrine usage	2,872 (46.38)	19,067 (46.55)	0.806	2,872 (46.38)	2,934 (47.38)	0.264
**1**st **trimester**	120 (1.94)	915 (2.23)	0.139	120 (1.94)	143 (2.31)	0.152
**2**nd **trimester**	1,494 (24.13)	9,404 (22.96)	0.042*	1,494 (24.13)	1,511 (24.40)	0.722
**3**rd **trimester**	1881 (30.38)	12,126 (29.60)	0.214	1881 (30.38)	1830 (29.55)	0.317
Ritodrine usage days	9.23 (11.19)	7.91 (9.43)	<0.001*	9.23 (11.19)	8.66 (10.67)	0.047*
Nifedipine usage	582 (9.40)	3,534 (8.63)	0.045*	582 (9.40)	558 (9.01)	0.456
**1**st **trimester**	36 (0.58)	236 (0.58)	0.960	36 (0.58)	41 (0.66)	0.568
**2**nd **trimester**	244 (3.94)	1,534 (3.75)	0.452	244 (3.94)	236 (3.81)	0.710
**3**rd **trimester**	391 (6.31)	2,243 (5.48)	0.007*	391 (6.31)	349 (5.64)	0.111
Nifedipine usage days	8.09 (10.11)	7.66 (11.60)	0.359	8.09 (10.11)	6.74 (7.30)	0.010*
Drug-supply days of tocolytics	12.73 (14.78)	10.03 (11.96)	<0.001*	12.73 (14.78)	11.01 (12.86)	<0.001*
Period between pregnancy to first tocolytic drugs usage	100.7 (65.63)	106.7 (69.77)	<0.001*	100.7 (65.63)	104.6 (68.31)	0.001*
Categories of tocolytic drugs usage			<0.001*			<0.001*
1	4,435 (71.62)	31,321 (76.47)		4,435 (71.62)	4,589 (74.11)	
2	1,566 (25.29)	8,833 (21.56)		1,566 (25.29)	1,463 (23.63)	
3	191 (3.08)	807 (1.97)		191 (3.08)	140 (2.26)	
Progestin and other tocolytic drugs			<0.001*			<0.001*
Progestin only	3,056 (49.35)	20,115 (49.11)		3,056 (49.35)	2,997 (48.40)	
tocolytic drug only	1,506 (24.32)	12,154 (29.67)		1,506 (24.32)	1749 (28.25)	
Progestin + tocolytic drug	1,630 (26.33)	8,692 (21.22)		1,630 (26.33)	1,446 (23.35)	
CHP usage days	23.72 (36.25)			23.72 (36.25)		
When to first use CHP						
1st trimester	4,845 (78.25)			4,845 (78.25)		
2nd trimester	400 (6.46)			400 (6.46)		
3rd trimester	947 (15.29)			947 (15.29)		
Birth outcome			<0.001*			0.075
Normal delivery	5,611 (90.62)	36,381 (88.82)		5,611 (90.62)	5,570 (89.95)	
Preterm birth	411 (6.64)	3,605 (8.80)		411 (6.64)	471 (7.61)	
Abortion	136 (2.20)	736 (1.80)		136 (2.20)	112 (1.81)	
Stillbirth	34 (0.55)	239 (0.58)		34 (0.55)	39 (0.63)	

Abbreviations: PS, propensity score; WM: western medicine; CHP: Chinese herbal products.

*p*-values are obtained by Chi-square test or independent sample *t*-test. *p < 0.05.

Table 2 demonstrated the OR of birth outcomes of the two groups, compared with the WM group before PS matching the proportion and the adjusted OR of preterm birth in the WM/CHP group was significantly lower (OR: 0.80, 95% CI: 0.71–0.89). After PS matching and adjusting for maternal age, the categories of tocolytic drugs used, and cumulative tocolytic drug use days, the adjusted OR of the WM/CHP group (n = 411, 6.64%) remained significantly lower than that in the WM group (n = 471, 7.61%) (OR: 0.86, 95% CI: 0.74–0.99). However, there were 136 (2.20%) pregnant women in WM/CHP and 112 (1.81%) pregnant women in WM group suffered from miscarriage, respectively. There was no significant difference in the incidence of miscarriage between the two groups (OR: 1.38, 95% CI: 0.93–2.05).

**TABLE 2:** Odd ratio estimates for birth outcome (preterm labor or miscarriage) of pregnant women between WM and WM/CHP groups.

	Number of Miscarriage	OR^a^	95% CI	*p*-value
*Before PS match*				
WM group	736	1		
WM/CHP group	136	1.09	0.89–1.33	0.404
*After PS match*				
WM group	112	1		
WM/CHP group	136	1.38	0.93–2.05	0.107
-	Number of preterm labor	OR^a^	95% CI	*p*-value
*Before PS match*				
WM group	3,605	1		
WM/CHP group	411	0.80	0.71–0.89	<0.001*
*After PS match*				
WM group	471	1		
WM/CHP group	411	0.86	0.74–0.99	0.040*

Abbreviations: OR, odd ratios; PS, propensity score; WM: western medicine; CHP: Chinese herbal products.

a Conditional logistic regression model adjusted for status of pregnancy age, categories of tocolytic drug usage, and cumulative tocolytic drugs usage days.*p < 0.05.

Table 3 presented the subgroup analysis based on the use of activating blood and removing stasis formulae or purgative formulae, the adjusted OR of preterm birth of those using these formulae was significantly lower in the WM/CHP group (n = 215) than that in the WM group (n = 265) (OR: 0.79, 95% CI: 0.65–0.96). In contrast, there was no significant difference between the WM/CHP and WM groups in the incidence of miscarriage or preterm birth among pregnant women not receiving blood activating and stasis removing formula or purgative formula. Furthermore, we also listed the top five single herb and herbal formulae in Supplementary Table S1, S2. (The composition and indications are derived from the classical herbal pharmacopeia).

**TABLE 3:** Subgroup analysis for odd ratios of birth outcome (preterm labor or miscarriage) based on treated with activating blood and removing stasis formulae or purgative formulae between WM and WM/CHP groups.

	Number of Miscarriage	OR^a^	95% CI	*p*-value
Non-treated with activating blood and removing stasis formulae or purgative formulae				
WM group (n = 2,472)	49	1		
WM/CHP group (n = 2,483)	59	1.91	1.00–3.66	0.050
Treated with activating blood and removing stasis formulae or purgative formulae				
WM group (n = 3,210)	63	1		
WM/CHP group (n = 3,264)	77	1.17	0.69–1.97	0.560
-	Number of preterm labor	OR^a^	95% CI	*p*-value
Non-treated with activating blood and removing stasis formulae or purgative formulae				
WM group (n = 2,629)	206	1		
WM/CHP group (n = 2,620)	196	0.97	0.78–1.20	0.769
Treated with activating blood and removing stasis formulae or purgative formulae				
WM group (n = 3,412)	265	1		
WM/CHP group (n = 3,402)	215	0.79	0.65–0.96	0.016*

Abbreviations: OR: odd ratios; WM: western medicine; CHP: Chinese herbal products.

a Conditional logistic regression model adjusted for status of pregnancy age, cumulative tocolytic drugs usage days and propensity score. *p < 0.05.

## 4 Discussion

To the best of our knowledge, this was the first study to conduct a correlation assessment of perinatal outcomes between the use of tocolytic drugs and CHPs in a retrospective cohort of pregnant women who experienced threatened abortion in Taiwan, using the NHIRD. Although the cause of most miscarriages is unclear, they are presumably due to complex interactions between parental age, genetic, hormonal, immunological, comorbidities, and environmental factors (Garrido-Gimenez and Alijotas-Reig, 2015). Therefore, we included maternal age at delivery, comorbidities of major pregnancy-related diseases, and the use of CHP before pregnancy as variables in the statistical analysis and matched these to reduce the risk assessment that might affect outcome results. We found that the adjusted OR of preterm birth in the WM/CHP group was significantly lower than that in the WM group. Some women with symptoms of threatened abortion or preterm labor in Taiwan also use CHM for tocolytic treatment or to alleviate the side effects of treatment with WM tocolytic drugs. Therefore, co-treatment with CHM products may also reduce the side effects of tocolytic drugs and achieve better effects. Previous systemic reviews have reported that the proportions of patients in the WM/TCM and WM groups who experienced an extension of gestational age to 28 weeks (7 months) were 94.4% and 73.6%, respectively (Li et al., 2012). The meta-analysis results showed that the combination of TCM preparations and WM was more effective than either treatment strategy was alone (Li et al., 2014).

The incidence of threatened abortion is approximately 10%–25% and over half of these pregnant women will eventually experience miscarriage (Everett, 1997; Hossain et al., 2007). Most threatened miscarriages occur in the first trimester, and when threatened abortion occurs before gestation week 6, the incidence of miscarriage is as high as 29% (Basama and Crosfill, 2004; Mulik et al., 2004). Our results also showed that the first time tocolytic drugs were used was in the first trimester (nearly 60%–70%), and nearly 85% of the users were diagnosed with threatened abortion, including approximately 50% who used progesterone with other tocolytic drugs. Tocolytic therapy is generally used for the suppression of preterm labor or threatened abortion. Progesterone supplementation is the most commonly used treatment for preventing miscarriage. The results of systematic reviews suggest that progesterone-based derivatives are probably effective in the treatment of threatened miscarriage but may have little or no effect on the rate of preterm birth (Coomarasamy et al., 2011; Lee et al., 2017; Wahabi et al., 2018). However, some studies have reported that the use of progesterone therapy in the first trimester of pregnancy does not show tocolytic effects against miscarriages in women with a history of unexplained recurrent miscarriages (Coomarasamy et al., 2015). However, this recent clinical evidence of the efficacy of progesterone and progesterone-based derivatives remains controversial.

Progesterone is one of the earliest known mediators of embryo implantation and uterine quiescence. Additionally, progesterone has anti-inflammatory properties, which prevent prostaglandin production and inhibit human oxytocin receptor signaling (Younger et al., 2017). A wide variety of tocolytic drugs have been used to suppress uterine contractions as acute tocolytic therapy and maintenance, but these drugs usually have some unwanted reactions or adverse side effects in pregnant women. Calcium channel blockers (such as nifedipine) are antagonists that interfere with calcium ion transfer through the myometrial cell membrane to decrease intracellular free calcium concentration and promote myometrial relaxation.

A Cochrane Database review meta-analysis published in 2014 reported that calcium channel blockers showed benefits over betamimetics in prolongation of pregnancy and preventing serious neonatal morbidity and maternal adverse effects (Flenady et al., 2014). Selective β2 agonists (such as ritodrine) have been used in clinical practice for preterm labor since the 1980s, and are the only US Food and Drug Administration (FDA) approved drug for acute treatment of preterm labor, which is currently unavailable in the US. These drugs decrease intracellular cyclic AMP concentration and facilitate myometrial relaxation.

Randomized controlled trials comparing betamimetics with placebo have shown that betamimetics decreased the number of women in preterm labor who gave birth within 48 h (Neilson et al., 2014). However, the lack of benefit for long-term tocolysis and perinatal mortality is compounded by reports of an enhanced maternal morbidity rate (Li et al., 2005; EMA, 2013; Neilson et al., 2014). Because the effects of WM tocolysis are uncertain and divergent and few side effects will occur, it is necessary to regularly monitor the mother and fetus.

The five most prescribed single herbs identified in the present study were *Scutellaria baicalensis* Georgi (Huang Qin), *Eucommia ulmoides* Oliv. (Du Zhong), *Cuscuta chinensis* Lam. (Tu Si Zi), *Cyperus rotundus* L. (Xiang Fu), and *Atractylodes macrocephala* Koidz. (Bai Zhu) (Supplementary Table S1). The main ingredient in *Scutellaria baicalensis* Georgi is baicalein, and its effects may be mediated by the reduction of interferon (IFN)-γ and the increase of progesterone levels. These changes have a protective effect on injured decidual cells and increase blood supply to the placenta, thereby exerting anti-abortifacient effects (Ma et al., 2009; Wang et al., 2014).

*Atractylodes macrocephala* Koidz. (Bai Zhu) is used in combination with *Scutellaria baicalensis* Georgi (Huang Qin) to prevent miscarriage by suppressing maternal-fetal interface immunity (Zhong et al., 2002), while *Atractylodes macrocephala* Koidz. (Bai Zhu) also increases the availability of free radical scavengers and antioxidants and inhibits uterine contractions (Han et al., 2016; Wang et al., 2016). Previous study has shown that *Eucommia ulmoides* Oliv. (Du Zhong) contains isoflavonoids, which have been reported to exhibit phytoestrogenic and androgenic properties that may be related to the optimization of sex hormone activity in the maternal body (Hussain et al., 2016).

The main active ingredients of *Cuscuta chinensis* Lam. (Tu Si Zi) are flavonoids, which can increase estrogen receptor expression in the hippocampus, hypothalamus, and pituitary as well as luteinizing hormone receptor expression in the ovaries (Ke and Duan, 2013). Recent studies have reported that these flavonoids can promote the migration and invasion of extravillous trophoblast cells by increasing matrix metalloproteinase 9 (MMP9) expression and prevent miscarriage by activating Notch, AKT, and mitogen-activated protein kinase (MAPK) signaling pathways (Gao et al., 2018). Previous research has shown that these herbs also nourish the kidneys and have similar effects to those of endocrine hormones by acting directly on the ovaries and regulating the hypothalamic-pituitary-ovarian axis to prevent miscarriage (Zhu et al., 2014).

The top three herbal formulae used by pregnant women in the present study were Dang-Gui-Shao-Yao-San, Jia-Wei-Xiao-Yao-San, and Wen-Jing-Tang. Dang-Gui-Shao-Yao San is used to treat pregnancy-induced abdominal pain and blood deficiency syndrome. Recent clinical trials and animal experiments have found that it corrects luteal phase insufficiency via an antioxidant mechanism and antagonism of both prostaglandin F2-α and acetylcholine-induced uterine contractions (Usuki et al., 2002; Shen et al., 2005). Jia-Wei-Xiao-Yao-San can be used to treat premenstrual tension, climacteric syndrome, and infertility in women (Washio, 2003).

In particular, the anxiolytic effects of Jia-Wei-Xiao-Yao-San are considered mediated by neurosteroid synthesis, followed by stimulation of central gamma-amino-butyric acid A/benzodiazepine receptors via attenuation of stress-induced upregulation of α-synuclein and corticosterone and downregulation of protein phosphatase 2A in the hippocampus (Cao et al., 2016). Wen-Jing-Tang is effective in improving endocrine-related conditions in the treatment of disturbances of ovulation and may suppress contractions of the uterine smooth muscle (Hsu et al., 2003; Ushiroyama et al., 2006). Wen-Jing-Tang plus Dang-Gui-Sha-Yao-San was the most prescribed dual-herbal formula used to treat female infertility in Taiwan (Hung et al., 2016).

We further analyzed whether TCM herbs used for activating blood and removing stasis or as purgatives, which are contraindicated in pregnancy in TCM because they may cause adverse reactions leading to miscarriage, may actually be beneficial. The results showed that the odds of premature labor in the WM/CHP group were significantly lower than those in the WM group. Modern research has confirmed that some habitual miscarriage cases are associated with increased blood viscosity and possibly antiphospholipid antibodies (APA; i.e., anti-cardiolipin antibodies) that induce thrombosis in placental blood vessels with uterine spiral arterials and atherosclerosis (Pritchard et al., 2016; Santos et al., 2017). A recent systemic review indicated that some Chinese herbal medicines such as mild blood moving herbs used alone or in combination with progesterone-based western therapies may in fact help preserve pregnancy (Yang et al., 2013). This is particularly applicable to women with APA syndrome who have an increased risk of thromboembolic events during pregnancy (Zeng and Yao, 2010). Recent studies indicate that Gui-Zhi-Fu-Ling-Wan possesses significant spasmolytic effects on uterine tetanic contractions (Sun et al., 2016). Furthermore, some blood activating and stasis removing herbs can dilate blood vessels and inhibit platelet aggregation, thereby preventing the formation of thrombosis or blood coagulation and modulating T-cell function (Liu et al., 2013). Additional research is required in this area of reproductive medicine in the future.

This study has the following limitations. First, not all tocolytic drugs are covered by health insurance. Medication information such as whether pregnant women are required to use self-funded tocolytic drugs or TCM herbs at hospitals or the use of other herbs or health foods obtained from herbal clinics or pharmacies is not available. This lack of information leads to an underestimation of drug exposure. Access to information on interfering factors affecting outcome results such as being bedridden, smoking, living habits, occupation, and multiple births is limited by the database and cannot be obtained or controlled. Second, the dosing days provided in the database are the number of days the doctor prescribed the drug, which does not reflect actual use by the patient. In future studies, the definition of drug use in the WM/CHP group should be more protracted, the timing of drug use should be further considered, and this information should be used simultaneously or sequentially.

## 5 Conclusion

In conclusion, 13.1% of pregnant women use CHP and tocolytic drugs in combination during pregnancy and the OR of preterm birth in the WM/CHP group was significantly lower than that in the WM group. In the future, rigorous research is needed to investigate the causal relationships of our findings and the safety of the CHP used by pregnant women with threaten miscarriage.

## Data Availability

The datasets presented in this article are not readily available because datasets are restricted and not publicly available due to confidentiality agreements. Requests to access the datasets should be directed to H-CW, xuang@ms65.hinet.net.
