# Supplementary material for: Chinese Herbal Medicines Have Potentially Beneficial Effects on the Perinatal Outcomes of Pregnant Women
Source: Front Pharmacol. 2022 Jun 6;13:831690. doi: 10.3389/fphar.2022.831690 (PMC9207412; doi:10.3389/fphar.2022.831690)
Supplement: Supplementary file 1 [file DataSheet1.docx]

Supplementary Table 1. Top five single herb for pregnant women treated with CHM

| Formula ingredients (Single Herb ^a^) | | | | | |
| --- | --- | --- | --- | --- | --- |
| Pin-yin name  Chinese name | Latin name | Plant species | Indications | Frequency of prescription | |
|  |  |  |  | N | % |
| Huang Qin  黃芩 | *Scutellaria baicalensis* Georgi | S. baicalensis | 1、Clearing heat and expelling damp  2、Purging fire and detoxification  3、Cooling blood and arresting bleeding | 2271 | 4.57 |
| Du zhong  杜仲 | *Eucommia ulmoides* Oliv. | E. ulmoides | 1、Nourishing liver and kidney  2、Coordinating Chong and Conception Vessels  3、Tonifying kidney for preventing miscarriage | 1975 | 3.97 |
| Tu Si Zi  菟絲子 | *Cuscuta chinensi*s Lam. | C. chinensis | 1、Warming kidney and strengthening spleen  2、Tranquilizing fetus to prevent miscarriage | 1937 | 3.9 |
| Hsiang Fu  香附 | *Cyperus rotundus* L. | C. rotundus | 1、Dispersing stagnated liver qi  2、Regulating menstruation and reliefing pain | 1885 | 3.79 |
| Bai-Zhu  白朮 | *Atractylodes macrocephala* Koidz. | A. macrocephala | 1、Invigorating qi and strengthening the spleen  2、Drying dampness and promoting urination | 1551 | 3.12 |

Supplementary Table 2. Top five herbal formulae for pregnant women treated with CHM

| Formula Ingredients (Herbal Formulae ^b^) | | | | | | |  |
| --- | --- | --- | --- | --- | --- | --- | --- |
| Pin-yin name  Chinese name | Composition | Indications | | Frequency of prescription N % | | | |
| Dang-Gui-Shao-Yao-San  當歸芍藥散 | *Angelica sinensis* (Oliv.) Diels (Dang-Gui)  *Ligusticum chuanxiong* Hort. (Chuan-Qiong)  *Paeonia lactiflora* Pall. (Bai-Shao)  *Poria cocos* (Schw.) Wolf (Fu-Ling)  *Angelica dahurica* (Fisch. ex Hoffm.) Benth. et Hook. (Bai-Zhi)  *Alisma plantago-aquatica* subsp. *orientale* (Sam.) Sam. (Ze-Xie) | Deficiency of liver-yin and Blood stagnation  Spleen deficiency and damp stagnation  Menstrual disorders | 2942 | | 9.19 |  |  |
| Jia-Wei-Siao-Yao-San  加味逍遙散 | *Angelica sinensis* (Oliv.) Diels (Dang-Gui)  *Angelica dahurica* (Fisch. ex Hoffm.) Benth. et Hook. (Bai-Zhi)  *Paeonia lactiflora* Pall. (Bai-Shao)  *Bupleurum chinense* DC. (Chai Hu)  *Poria cocos* (Schw.) Wolf (Fu-Ling)  *Glycyrrhiza uralensis* Fisch. ex DC. (Gan-Cao)  *Paeonia suffruticosa* Andrews (Mu Dan Pi)  *Gardenia jasminoides* J.Ellis (Zhi Zi)  *Zingiber officinale* Roscoe (Sheng-Jiang)  *Mentha haplocalyx* Briq. (Bo He) | Liver depression  Disquieted fearful throbbing  Blood deficiency and fever menstrual irregularities | 2036 | | 6.36 |  |  |
| Wen-Jing-Tang  溫經湯 | *Tetradium ruticarpum* (A. Juss.) T.G. Hartley (Wu Zhu Yu)  *Panax ginseng* C.A.Mey. (Ren Shen)  *Cinnamomum cassia* (L.) J.Presl (Gui-Zhi)  *Ligusticum chuanxiong* Hort. (Chuan-Qiong)  *Zingiber officinale* Roscoe (Sheng-Jiang)  *Pinellia ternata (Thunb.) Makino* (Ban Xia)  *Glycyrrhiza uralensis* Fisch. ex DC. (Gan-Cao)  *Angelica sinensis* (Oliv.) Diels (Dang-Gui)  *Paeonia lactiflora* Pall. (Bai-Shao)  *Equus asinus L*.(A Jiao)  *Moutan officinalis* (L.) Lindl. & Paxton (Mu-Dan-Pi)  *Ophiopogon japonicus (Thunb.)* Ker Gawl. (Mai Men Dong) | Deficient cold of thoroughfare and conception vessels  Metrostaxis with syndrome of internal blockade of static blood | 1321 | | 4.13 |  |  |
| Bao-Chan-Wu-You-Fang  保產無憂方 | *Angelica sinensis* (Oliv.) Diels (Dang-Gui)  *Ligusticum chuanxiong* Hort. (Chuan-Qiong) *Cuscuta* *chinensis* Lam. (Tu Si Zi) *Paeonia lactiflora* Pall. (Bai-Shao)  *Fritillaria cirrhosa* D.Don, (Bei Mu)  *Astragalus propinquus* Schischkin (Huang Qi)  *Schizonepeta* *tenuifolia* (Benth.) Briq. (Jing Jie) *Artemisia* *argyi* H.Lév. & Vaniot (Ai Ye) [*Magnolia* *officinalis* Rehder & E.H.Wilson](http://www.theplantlist.org/tpl/record/kew-117741) (Hou Pu)  [*Citrus* *aurantium* L.](http://www.theplantlist.org/tpl/record/tro-28100388) (Zhi Ke)  *Notopterygium incisum* K.C.Ting ex H.T.Chang (Qiang-Huo)  *Zingiber officinale* Roscoe (Sheng-Jiang)  *Glycyrrhiza uralensis* Fisch. ex DC. (Gan-Cao) | Qi stagnation or blood stasis | 1190 | | 3.72 |  |  |
| Xiang-Sha-Liu-Jun-Zi-Tang  香砂六君子湯 | *Aucklandia lappa* DC.( Mu Xiang)  *Amomum villosum* Lour. (Sha Ren)  *Pinellia ternata (Thunb.)* Makino (Ban Xia)  *Citrus reticulata* Blanco (Chen Pi)  *Panax ginseng* C.A.Mey. (Ren Cen)  *Atractylodes macrocephala* Koidz, (Bai-Zhu)  *Poria cocos* (Schw.) Wolf (Fu-Ling)  *Glycyrrhiza uralensis* Fisch. ex DC.(Gan-Cao) | Deficiency of spleen and stomach  Damp obstruction and qi stagnation | 1074 | | 3.35 |  |  |

^a^ The total number of prescriptions for single herb is 49,693; ^b^ The total number of prescriptions for herbal formulae is 32,015

* The composition and indications of the herbal formulae are derived from the reference of the Taiwan Herbal Pharmacopeia 2nd Edition English version.
